# Biochemical Characterization of New Sweet Orange Mutants Rich in Lycopene and β-Carotene Antioxidants

**DOI:** 10.3390/antiox13080994

**Published:** 2024-08-16

**Authors:** Lourdes Carmona, Berta Alquézar, Leandro Peña

**Affiliations:** 1 Instituto de Biología Molecular y Celular de Plantas, Consejo Superior de Investigaciones Científicas, Universidad Politécnica de Valencia, 46022 Valencia, Spain; lcarmona@ibmcp.upv.es (L.C.); beralgar@ibmcp.upv.es (B.A.); 2 Fundo de Defesa da Citricultura (Fundecitrus), Araraquara 14807-040, SP, Brazil

**Keywords:** carotenoids, lycopene, β-carotene, red-fleshed oranges, antioxidants, phytonutrients

## Abstract

Carotenoid-rich foods such as citrus fruits have a wide range of functions in human health. They primarily exert antioxidant effects, but individual carotenoids may also act through other health-promoting mechanisms such as β-carotene as pro-vitamin A. Here, we show that red-fleshed sweet oranges grown in tropical climates are 4–9 times richer in carotenoids than their orange-fleshed counterparts, regardless of their maturation stage. The most significant difference observed between both varieties was the presence of lycopene at moderate concentrations (around 8 µg/g FW) in the mature pulp of the red varieties, which was absent in the blond ones. This is because the red-fleshed sweet oranges grown in tropical climates with high temperatures increase lycopene and β-carotene concentrations in their pulp during fruit maturation. Due to lycopene accumulation, red orange juice offers a promising addition to popular blond-orange, with the new varieties Carrancas and Pinhal being perfectly suitable for blending to enhance juice colour. Sao Paulo, one of the world’s leading citrus orange juice producers, as well as other tropical citrus regions could benefit from cultivating using such lycopene-rich cultivars and industrially.

## 1. Introduction

Carotenoids are a large family of isoprenoid pigments that have essential functions as components of the light-harvesting system and in protecting plant cells against oxidative processes [1]. These pigments are also components of the human diet, with important antioxidant activity and protective effects against carcinogenesis, cardiovascular diseases and degenerative progression [2,3,4,5,6]. Moreover, carotenoids with at least one unsubstituted β-ionone ring like β-carotene or β-cryptoxanthin are the precursors of vitamin A [7,8,9].

Carotenoids impart attractive colours to many fruits and vegetables, including citrus fruits [10,11]. Citrus fruit and juice are widely consumed, with a total production of 158.5 million tons of fruit per year worldwide [12], positioning them as important sources of carotenoids for the human diet [13,14]. The *Citrus* genus displays a complex carotenoid profile, with more than 110 different carotenes and xanthophylls reported, provide external and internal fruit coloration of the main citrus species and hybrids [15,16,17]. The carotenoid content and composition impact both the commercial and nutritional quality, these are the major determinants of consumer acceptance, since the external colour of citrus fruit and juice constitutes the consumer’s first perception of the product and thus represents a primary quality attribute. Differences in the carotenoid profiles in mature citrus fruits, where content and composition vary depending on the cultivar [17,18], are responsible for the wide range of colorations that can be found among diverse citrus cultivars, from the white of grapefruits, pale yellow of some lemons, the intense orange of certain sweet oranges and mandarins to the red of specific oranges and grapefruits [16]. For example, although the β,β-xanthophyll violaxanthin predominates in mandarin and sweet orange fruits, a higher β-cryptoxanthin content can be found in the former, providing their distinctive orange-reddish colour [16]. On the other hand, lemons are characterised by an accumulation of linear colourless carotenes (see the review in [17]).

**Figure 1:**
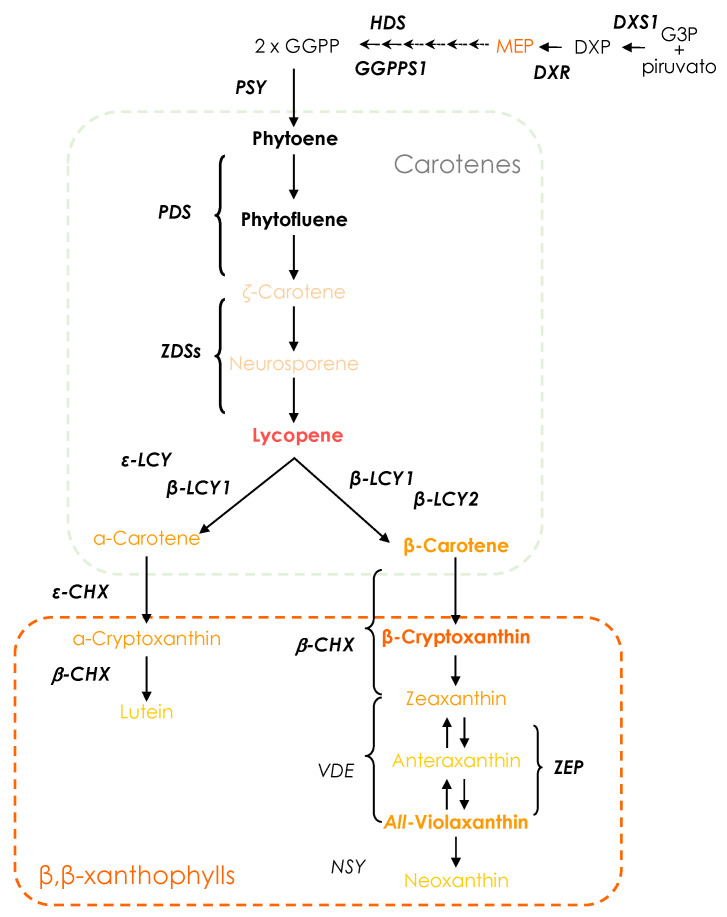
Schematic diagram of the biosynthesis pathway of carotenoid in citrus fruit. The carotenoids and genes analysed in this work are bold-lettered. G3P, glyceraldehyde-3-phosphate; DXP, deoxyxylulose 5-phosphate; MEP, methyl-D-erythritol 4-phosphate; GGPP, geranylgeranyl pyrophosphate; DXS, DXP synthase; DXR, DXP reductoisomerase; HDS, HDR, hydroxymethylbutenyl 4-diphosphate reductase; GGPPS, geranylgeranyl pyrophosphate synthase;PSY, phytoene synthase; PDS, phytoene desaturase; ZDS, ζ- carotene desaturase; ε-LCY, lycopene ε-cyclase; β-LCY1, lycopene β-cyclase 1; β-LCY2, lycopene β-cyclase 2; β-CHX, β-carotene hydroxylase; εCHX, ε-carotene hydroxylase; ZEP, zeaxanthin epoxidase; VDE, violaxanthin de-epoxidase; NSY, neoxanthin synthase. Metabolites are coloured according to their colours, whereas black indicates no colour. Dotted rectangles separate different groups of carotenoids.

The carotenoid biosynthesis pathway has been well characterised [17,19,20,21,22]. Carotenoids are derived in light-grown plants from the precursor isopentenyl diphosphate (IPP) and its isomer dimethylallyl diphosphate (DMAPP) from the methyl-D-erythritol 4-phosphate (MEP) pathway, which occurs in the plastids [23,24]. The first step is catalysed by *DXP synthase* (*DXS*) to produce deoxyxylulose 5-phosphate (DXP), which is reduced by *DXP reductoisomerase* (*DXR*) and yields MEP (Figure 1). MEP is converted into the precursors IPP and DAMPP by the sequential action of different enzymes. The condensation of these precursors generates geranylgeranyl pyrophosphate (GGPP). Through the action of *phytoene synthase* (*PSY*), two GGPPs are converted into the colourless carotene named phytoene, which is desaturated to phytofluene by *phytoene desaturase* (PDS). Phytofluene is desaturated by *ζ-carotene desaturase* (ZDS), generating the red-coloured carotene lycopene, which is cyclised by *lycopene ε-cyclase* (*εLCY*) and *lycopene β-cyclase* (*βLCY*) to produce α-carotene or by the enzyme *βLCY* to generate β-carotene. In orange fruits, the accumulation of carotenoids during the maturation process is coordinated by the induction of *βLCY2* expression, concomitantly with a decrease in *εLCY* expression, favouring a shift to β,β-xanthophylls production. The *β-carotene hydroxylase* (*βCHX*) enzyme acts on β-carotene, yielding zeaxanthin via β-cryptoxanthin, followed by the epoxidation reaction of zeaxanthin via *zeaxanthin epoxidase* (*ZEP*) to generate violaxanthin, the most abundant carotenoid in sweet orange juices.

In rare case, citrus fruits accumulate lycopene. This has been reported and characterised in only a few species, such as grapefruits (*Citrus paradisi* Marcf.) and pummelo (*Citrus grandis* Osbeck) [25]. Lycopene is a potent antioxidant carotene which confers major nutritional and nutraceutical value [26,27]. Few sweet oranges have also been reported to be capable of accumulating lycopene, such as Shara orange, a sport of Shamouti [28], the red orange Hong Anliu, a bud mutation of Anliu sweet orange [29] and Cara cara, which originated in Venezuela as a bud mutation from a Navel orange [25,30]. In addition to the nutritional value of red oranges, lycopene accumulation provides an atypical internal colouration that varies from the pink of the Shara orange to the bright red pulp of Cara cara oranges, providing important commercial value to these varieties. Because of that, the main citrus industries have been focused on searching for and selecting sweet orange types containing lycopene pigmentation in the recent years, including Puka Valencia from Argentina or Ruby Valencia from South Africa [31]. Sao Paulo (Brazil), which contributes 70% of worldwide juice production, has incorporated Puka and Cara cara red-oranges as commercial cultivars of interest [32]. More recently, the red orange Mombuca has been also selected, this is a Brazilian red variety that originated as a spontaneous sweet orange mutant [33]. The characterisation of new red-orange cultivars could offer a strategy for the citrus industry to extend the range of colours and increase the nutritional properties of orange juice. Thus, the aim of this work was to characterise the fruit quality and biochemical features of two new red-fleshed sweet orange spontaneous mutants, Carrancas and Pinhal, both originated and selected in Sao Paulo (Brazil). It is presented here that both new mutants are productive, mid-season varieties, showing high accumulation of lycopene and β-carotene phytonutrient carotenoids, with sufficiently high juice quality to consider them as possible new appealing red-flesh additions to the currently existing and highly successful blond-orange varieties.

## 2. Materials and Methods

### 2.1. Plant Material

In this study, sweet orange (*C. sinensis* L. Osbeck) fruits from cultivars Hamlin (Ha), Pera (Pe) and Valencia (Va), were used as early, mid-season and late-season blond oranges, respectively, Cara cara (Cc), Carrancas (Ca), Mombuca (Mo), Pinhal (Ph) and Puka (Pk) were used as early (Cc), mid-season (Ca, Mo, Ph) and late (Pk) red-fleshed mutants, at different developmental stages: immature-green (IG), mature-green (MG), breaker (BR) and full-coloured (FC). They were harvested at random from adult (4-year-old) trees grown under standard conditions in an experimental area in a commercial orchard (Fazenda Guacho) in Sta. Cruz de Rio Pardo, Sao Paulo State, Brazil (22°53′56″ S–49°37′58″ O) during two consecutive seasons (season 1: 2021/22–season 2: 2022/23). The climatological conditions of the area were monitored (Figure S1). Fruits were uniform in size and colour, and free of damage or defects. Pulp was separated with a scalpel, immediately frozen in liquid nitrogen, ground to a fine powder and stored at −80 °C until analysis. For all analyses, 3 replicate samples of 5 fruits each per developmental stage were used.

### 2.2. Analysis of Fruit Quality

Fruit quality assessment was carried out for every cultivar over two seasons. Measurements of quality parameters were achieved based on fruit samples for every citrus tree from the orchard. A total of 40 fruits (4 samples of 10 fruits each) were harvested annually when the fruit was fully mature. The following fruit quality parameters were measured and averaged for each sample: fruit weight (W), fruit volume (V), fruit diameter (D), fruit height (H), cortex fruit thickness (C), peel fruit thickness (P), number of segments (Sg), number of seeds (S), juice content (JC), total soluble solids (TSS), titratable acidity (TA) and maturity index (MI). The parameters were measured as described in Pons et al. [34]. All measures are presented as the mean ± standard error of each sample.

### 2.3. Carotenoid Extraction and UPLC Analysis of Individual Carotenoids

Carotenoids were extracted as is described in Carmona et al. [35]. The carotenoid composition of each sample was analysed through UPLC with a Nexera X2 Shimadzu liquid chromatography system equipped with a LC-30AD pump and a SPD-M20A photodiode array detector, and LabSolution software (version 5.57 SP1). An Acquity BEH C18 carotenoid column (100 mm × 2.1 mm, 1.8 µm) coupled to a C18 guard column (20 mm × 2.1 mm) (Waters, Milford, MA, USA) was used. The samples were prepared for UPLC by dissolving the dried carotenoid extracts in CHCl_3_:MeOH:acetone (3:2:1, *v*:*v*:*v*). For carotenoid separation, a binary gradient elution was adapted for red oranges [36] from the ternary described by Alquezar et al. [37] for red-oranges by using the Gradient Method Calculator (Thermo Scientific, Waltham, MA, USA). Carotenoids were identified based on their retention time, absorption and fine spectra [38,39,40,41]. The carotenoid peaks were integrated at their individual maxima wavelength and their contents were calculated using calibration curves of β-carotene (Sigma, St. Louis, MO, USA) for α- and β-carotene; β-cryptoxanthin (Extrasynthese, Genay, France) for β-cryptoxanthin; zeaxanthin (Extrasynthese) for zeaxanthin; lutein (Sigma) for violaxanthin isomers, mutatoxanthin and antheraxathin; lycopene (Sigma) for lycopene and phytoene (Sigma) for phytoene and phytofluene. The total carotenoids were calculated as the sum of all quantified individual ones.

### 2.4. Total RNA Isolation and Quantitative RT-PCR Analysis

The total RNA extraction, DNase treatment, cDNA synthesis, quantitative real-time PCR procedure and primers used for the analysed genes followed Carmona et al. [42]. Th primer sequences used for each gene used are detailed in Table S1 [35,37,43,44,45]. To demonstrate the expression stability of the reference genes *glyceraldehyde-3-phosphate dehydrogenase C2* (*GAPC2*), *ubiquitin-conjugating enzyme 21* (*UBC21*) and *ubiquitin-protein ligase 7* (*UPL7*) under our experimental conditions, the algorithm geNorm was used (https://genorm.cmgg.be/, accessed on 1 February 2021) [46]. The relative expression of genes of interest (GOI) in this study was determined throughout the maturation process as described by Carmona et al., [42]. Values are presented as the mean of at least three independent analyses ± SD.

## 3. Results

### 3.1. Fruit Appearance at Different Developmental Stages and Fruit Quality Parameters of New Red-Fleshed Sweet Orange Mutants

**Figure 2:**
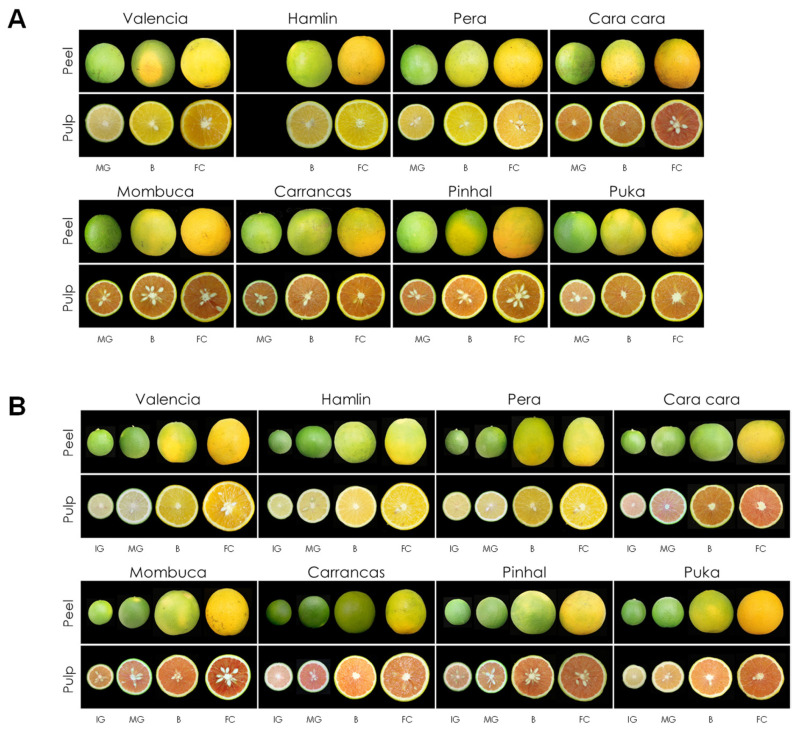
External and internal appearance of Valencia, Hamlin, Pera, Cara cara, Mombuca, Carrancas, Pinhal and Puka fruits (*C. sinensis* L. Osbeck) in two different seasons: (**A**) Season 1 (2021/2022) and (**B**) Season 2 (2022/2023). The physiological fruit stages are indicated: IG (Immature-green), MG (Mature-green), B (breaker) and FC (Full-color).

The changes in external and internal appearance during the maturation period for each variety are shown in Figure 2. Peel and pulp colour progressively turned from green to red or orange along the maturation process, with a remarkable treddish coloration occurring in the pulp of red-fleshed cultivars. The differences in pulp coloration between the blond cultivars Hamlim (Ha), Pera (Pe) and Valencia (Va), and the red-fleshed ones Cara cara (Ca), Mombuca (Mo), Carrancas (Ca), Pinhal (Ph) and Puka (Pk) were detected from the initial stages of fruit development. The pulp of immature-green (IG) fruit from blond cultivars displayed a yellow colour while fruit belonging to red mutants already presented a distinctive reddish-pink colouration at that developmental stage (Figure 2B). Then, during the maturation period, the internal colouration of Ha, Pe and Va fruit turned a characteristic orange colour (Figure 2). The red-fleshed cultivars remained deep red throughout their development, displaying orange shades in advanced mature stages. The intensity of the red colour at full maturity and their progress during maturation were comparable for all red mutants, with small variations depending on the season.

The fruit quality parameters of each sweet orange cultivar at the mature stage are represented in Table 1 The highest total soluble solids (TSS) were found in blond Pe and Va cultivars (12.6° ± 0.2° and 11.8° ± 0.3° Brix, respectively), while Mo and Ca presented the lowest TSS (8.5° ± 0.2° and 8.0° ± 0.4° Brix, respectively) in season 2. In general, TSS values were lower in season 1, although there remained differences among varieties. The titratable acidity (TA) was similar for most cultivars in both seasons, with Ca, Ph and Mo having the lowest acidity (0.4° ± 0.3°, 0.5° ± 0.0° and 0.5° ± 0.2°, respectively in 2022/2023). The Maturity index (MI) expressed as the ratio of TSS/TA and measured for each variety and season showed that blond varieties tended to display lower values (ranging from 14.5° ± 0.2° to 17.9° ± 0.5°, in season 2) due in part to increased acidity, while intermediate concentrations were found in the red-fleshed mutants Mo and Pk, which presented MIs of 17.8° ± 0.4° and 19.7° ± 0.4° in season 2. The red-fleshed Ca and Ph showed the highest MI ratios, which were around 19–20 for both seasons (Table 1).

### 3.2. Carotenoid Composition of Red-Fleshed Sweet Orange Pulp during Maturation

**Figure 3:**
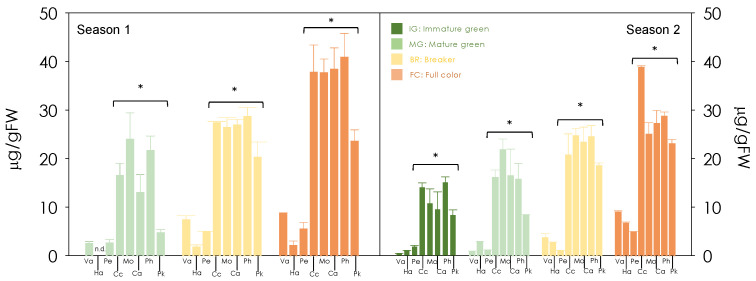
Changes in carotenoid contents in the pulp during development and maturation of Valencia (Va), Hamlin (Ha), Pera (Pe), Cara cara (Cc), Mombuca (Mo), Carrancas (Ca), Pinhal (Ph) and Puka (Pk) fruits (*C. sinensis* L. Osbeck) in two different seasons. The 10 samples analysed correspond to the developmental and maturation stages indicated in Figure 2. The physiological fruit stages are indicated: IG (immature-green), MG (mature-green), B (breaker) and FC (full-colour). The data are means ± SD of at least three independent measurements. Statistical analyses were performed using analysis of variance (ANOVA) and an asterisk above the bars indicates significantly different values at *p* ≤ 0.01 (n.d.: non detected).

**Figure 4:**
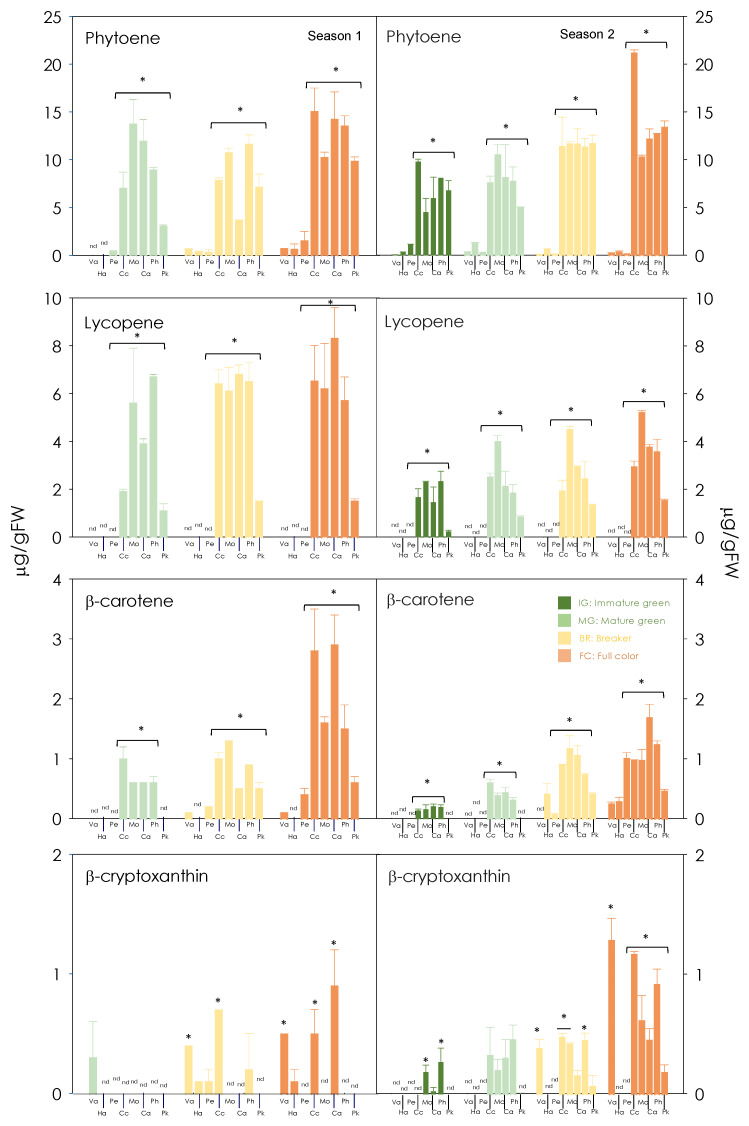
Carotenoid content of phytoene, phytofluene, β-carotene and β-cryptoxanthin in the pulp during development and maturation of Valencia (Va), Hamlin (Ha), Pera (Pe), Cara cara (Cc), Mombuca (Mo), Carrancas (Ca), Pinhal (Ph) and Puka (Pk) fruits (*C. sinensis* L. Osbeck) in two different seasons. The samples analysed correspond to the developmental and maturation stages indicated in Figure 2. The physiological fruit stages are indicated: IG (immature-green), MG (mature-green), B (breaker) and FC (full-colour). The data are means ± SD of at least three independent measurements. Statistical analyses were performed using analysis of variance (ANOVA) and an asterisk above the bars indicates significantly different values at *p* ≤ 0.01 (n.d.: non detected).

The total content and composition of carotenoids was analysed in the pulp of the three blond oranges and five red-fleshed mutants at four developmental stages: immature-green (IM), mature-green (MG), breaker (Br) and full-coloured (FC) (Figure 3, Figure 4 and Figure S2). Seven main carotenoids were measured through UPLC analysis, accounting for more than 90% of the total carotenoids in all samples analysed. The total carotenoid content was calculated as the sum of all carotenoids identified (Figure 3). The changes in total carotenoid content were markedly different in the blond oranges compared to red-fleshed mutants. In the blond cultivars, the total carotenoid content in the pulp was very little until the Br stage, and then increased to reach its maximum values of 4.8–8.9 µg/g FW and 2.0–8.6 µg/g FW in seasons 2 and 1, respectively. The carotenoids accumulation in the pulp of red-fleshed mutants was high from the early stages of development (8.3–15.1 µg/g FW and 4.6–23.9 µg/g FW in seasons 2 and 1, respectively) and progressively increased throughout the whole maturation process (23.0–38.7 µg/g FW and 25.5–40.8 µg/g FW in seasons 2 and 1, respectively). The pulp of the red-fleshed mutants at early stages presented about 10-times higher concentrations of total carotenoids than that of the blond cultivars. From the Br to FC stages, the total carotenoid content in the pulp of the red mutants was 4 to 9 times higher than that in the blond oranges.

The evolution of carotenoid content and composition in the pulp of blond oranges was markedly different to that of red-fleshed mutants, with differences obvious from the IG stage, well before the beginning of the maturing process (Figure 4 and Figure S2). During the maturation of blond oranges, the pulp accumulated almost exclusively xanthophylls, with *all*-violaxanthin (as the sum of *E*-violaxanthin and 9-*Z*-violaxanthin) as the major carotenoid, which accounted for more than 50% of the total carotenoids in mature fruit. On the contrary, linear carotenoids were predominant carotenoids in the pulp of all the red-fleshed mutants, accounting for more than 70% of total carotenoids in all stages analysed. Phytoene concentration augmented gradually as fruit matured, containing around 3–17 fold (0.8–9.8 fold) more than the pulp of the blond cultivars in season 2. A similar pattern was noticed for the other linear carotene phytofluene, whose content increased 1–9 fold (2.1–8.3 fold) in season 2 during maturation in red oranges. This carotenoid was not detected in blond fruits. For both compounds, Pk was the red-fleshed mutant with the least accumulation. As expected, no lycopene was detected in Ha, Pe and Va throughout all maturation stages and β-carotene only was detected in traces in mature stages. In the case of red-fleshed mutants, both bioactive carotenes were accumulated in the pulp from the early stages on. The maximum lycopene content was reached in mature fruit in all red-fleshed mutants, with Pk being the cultivar with the lowest content of these pigments in both seasons, 1.5 ± 0.5 and 2.2 ± 0.4 µg/g FW in season 2 and 1, respectively. Instead, the other red-fleshed mutants accumulated higher contents of lycopene, reaching up to 7.2 ± 1.5 µg/g FW or 8.3 ± 1.0 µg/g FW as was the case of the Cc and Ca varieties (Figure 4). There was a higher accumulation of β-carotene in red-fleshed mutants (between 0.6–2.9± 0.1 µg/g FW and 0.5–1.7 ± 0.2 µg/g FW for season 1 and 2, respectively) than blond cultivars (between 0.1–0.4 ± 0.1 µg/g FW and 0.2–0.1 ± 0.2 µg/g FW for season 1 and 2, respectively). In any case, the mutant Ca accumulated the highest quantity of β-carotene in both seasons (Figure 4). Related to carotenoids of the ε,β-branch, only traces of α-carotene and α-cryptoxanthin were detected in all the varieties during the maturation period, while lutein accumulated during the maturation process, with the exception of Va in which it was only was detected in the early stages (Figure 4 and Figure S2). The accumulation of β,β-xanthophylls (as the sum of β-cryptoxanthin, mutatoxanthin, anteraxanthin, *E*-violaxanthin and 9-*Z*-violaxanthin) was increased in the pulp along the maturation process in all varieties, presenting the highest contents in the Va cultivar, 8.5 ± 0.4 µg/g FW and 7.5 ± 0.1 µg/g FW in season 2 and 1, respectively (Figure 4 and Figure S2). At full maturation stage, red-flesh mutants presented a higher content of β,β-xanthophylls, being Cc and Pk showing the highest contents (4.1 ± 0.3 µg/g FW and 3.5 ± 0.3 µg/g FW in season 2 and 1, respectively).

### 3.3. Gene Expression Profile of Carotenogenic Genes during Development and Maturation in the Pulp of Red-Fleshed Sweet Oranges

**Figure 5:**
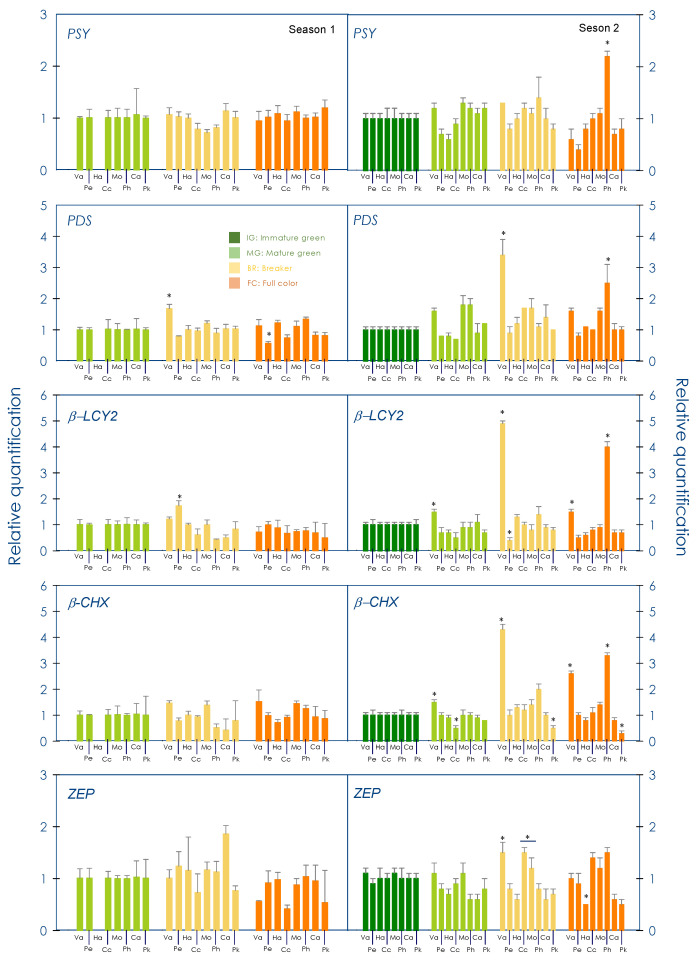
Quantitative RT-PCR analysis of the expression of PSY, PDS, β-LCY2 β-CHX and ZEP genes in the pulp during development and maturation of Valencia (Va), Hamlin (Ha), Pera (Pe), Cara cara (Cc), Mombuca (Mo), Carrancas (Ca), Pinhal (Ph) and Puka (Pk) fruits (*C. sinensis* L. Osbeck) in two different seasons. The samples analysed correspond to the developmental and maturation stages indicated in Figure 2. The physiological fruit stages are indicated: IG (immature-green), MG (mature-green), B (breaker) and FC (full-colour). Statistical analyses were performed using analysis of variance (ANOVA) and an asterisk above the bars indicates significantly different values at *p* ≤ 0.01.

In order to determine whether the carotenoid composition in red-fleshed oranges was associated with specific gene expression patterns, the expression of a total of 14 genes involved in carotenoid production was analysis (Figure 5). Four genes of the MEP pathway and eleven genes of the carotenoid pathway were analysed in the pulp of every cultivar at the different developmental stages, as shown in Figure 2. Transcript accumulation of *DXS* and *HDR* presented a maximum at the MG in season 1, although in season 2 it was similar to the FC stage, with the exception of the Ph cultivar which showed the highest expression (3.7 ± 0.3 and 3.7 ± 0.1, relative expression for *DXS1* and *HDR*, respectively) compared with other cultivars at this stage (Figure S3). In general, the *HDS* gene showed the lowest expression of all genes in the MEP pathway, while *GGPPS1* presented no changes during season 1 and only a slight increment in MG and FC stages in the second season (Figure S3).

The expression profile of the carotenogenic genes was different between seasons. While in season 1 no relevant changes were observed between the MG, Br and FC stages, several differences were detected in season 2 (Figure 5 and Figure S4). Among them, the Va cultivar displayed a boost in the expression of *PDS*, *β-LCY2* and *β-CHX* in the MG stage (3.4 ± 0.5, 4.9 ± 0.1 and 4.3 ± 0.2, respectively). On the other hand, Ph was the cultivar with the highest expression of carotenogenic genes in the FC stage with a relatively higher expression of 2.2 ± 0.1, 2.5 ± 0.6, 4.0 ± 0.2, 3.4 ± 0.1 and 2.0 ± 0.2, for *PSY*, *PDS*, *ZDS1*, β-*LCY2* and *β-CHX*, respectively (Figure 5 and Figure S3). In any case, these differences in gene expression did not explain the carotenoid profiles in the pulp in red-fleshed sweet oranges fruits compared to that of blond cultivars.

## 4. Discussion

There is currently an active search in most fruit industries worldwide for citrus fruit mutants with distinctive attractive colours, especially reddish hues. This is not only because of the appealing of the red colour for most human cultures [47,48] but also because it is associated with the accumulation of healthy phytonutrients, namely carotenoids such as lycopene. It is shown here that the juice and pulp from sweet orange varieties producing red-fleshed fruit are four to nine times richer in carotenoids than those from orange-fleshed counterparts when trees are grown under a tropical climate and independently of fruit maturation of early, mid-season and late varieties, reinforcing an association between red colour and the functional attributes of sweet orange fruit and juices. Carotenoid-rich foods have been associated with risk reduction for several chronic diseases, including reduced incidence of obesity, type 2 diabetes, eye diseases, certain types of cancer (especially prostate and digestive tract tumours), and even lower total mortality. In addition, some carotenoids, particularly β-carotene, constitute important vitamin A precursors [49,50]. Carotenoids have been widely recognised as physiological antioxidants because of their ability to quench singlet molecular oxygen [5,6,51].

The carotenoid profiles in the fruit pulp/juice showed that linear carotenoids such as phytoene and phytofluene were the most abundant in all the five red-fleshed varieties, representing more than 70% of total carotenoids in all developmental/maturation stages, while the blond varieties accumulated mainly xanthophylls, with *all*-violaxanthin depicting more than 50% of total carotenoids in mature fruit. Nevertheless, the most remarkable difference was the accumulation of lycopene at moderate concentrations (about 8 µg/g FW) in mature pulp from red fruit, while it was absent in blond varieties. Moreover, red-fleshed varieties accumulated β-carotene in mature fruit for at least one of the seasons while this compound was only detected in traces in blond varieties (Figure 3, Figure 4 and Figure S2). A positive association between the accumulation of linear carotenoids and lycopene has been described before for Cc and other red-fleshed sweet orange varieties [29,37,52,53] when grown under sub-tropical climate conditions, but not in the Chinese Anliu sweet orange variety, which does not accumulate upstream carotenes but has lycopene-rich pulp [29,54]. Such correlation was shown in the case of the five red-fleshed varieties studied here, suggesting that enhanced *PSY* activity may be responsible at least in part for the accumulation of lycopene [37] as it occurs in other fruits such as tomato [55,56]. Together with this or alternatively, a partial blockage in the function of *β-LCY2* has been proposed in other lycopene-rich citrus varieties [57,58]. However, the accumulation of lycopene and β-carotene was not accompanied by a consistent decrease in the production of xanthophylls (Figure 4 and Figure S2) as it has been reported in other works with red-fleshed sweet orange mutants [37,53] and as it may be expected if *β-LCY2* expression were interfered with. Moreover, we did not find alterations of the transcriptional profiles in the pulp of the red-fleshed oranges, neither in the expression of MEP pathway gene precursors of phytoene or in that of *β-LCY2*, when compared with those of the blond orange controls (Figure 5, Figures S3 and S4). Therefore, the molecular features behind the mutation that leads to the accumulation of lycopene in the pulp of these (and other) sweet orange varieties remain to be elucidated, although this mutation seems to be post-transcriptionally regulated at the level of the carotenoid biosynthetic pathway.

Seasonal differences were observed in the accumulation of carotenoids for all the varieties investigated here. For example, highest concentrations of lycopene and β-carotene in the pulp of red-fleshed varieties Cc, Mo, Ca and Ph were linked to higher maximum temperatures during fruit maturation in season 2 vs. season 1. Although arelatively low temperature is required for chlorophylls to degrade and for carotenoids to accumulate during the maturation of citrus fruits, it is well known that high temperatures in the later stages of fruit maturation enhance the accumulation of lycopene and other carotenoids in the pulp of lycopene-rich varieties (review in Alquezar et al. [37]). Our results indicate that growing lycopene-rich varieties in other areas of Sao Paulo state, at the north and centre of the region (Figure S1), closer to the main areas of the citrus belt, where temperatures are more amenable than in the south, may further improve the accumulation of lycopene and β-carotene.

So far, lycopene and β-carotene are the most investigated carotenoids in search for in vivo human health benefits [50,59]. Apart from the role of β-carotene as provitamin A precursor, recent epidemiological and cellular studies are showing the antioxidant effect of lycopene in inhibiting the development of diseases such as cardiovascular diseases inflammation, obesity, neurodegenerative disorders, type 2 diabetes, metabolic diseases that affect the bone, eye, kidney and liver and ulcerative colitis [26,27,60]. Moreover, lycopene accumulates at high concentrations in the testis, where its antioxidant activity may help to eliminate oxidative damage [59,60].

Sao Paulo state (Brazil) concentrates 70% of the orange juice produced worldwide. Ha, Pe and Va, used here as blond-orange controls, are the most important varieties grown in Sao Paulo, representing about 80% of the total orange juice produced. Lycopene-rich red orange juice may become an appealing addition to the already highly popular blond orange juices. Considering the five red-fleshed mutants investigated, Cc is not a suitable variety for the juice industry, because it accumulates the bitter triterpene compound limonin in its flesh just minutes after processing, like most Navel oranges [25]. Pk is a mutant from Va, and as suchis the only late red-fleshed variety of the group. However, it does accumulated the lowest concentrations of lycopene and β-carotene. From the three mid-season red-fleshed mutants, Mo showed the lowest MI and Ca showed the highest amounts of lycopene/β-carotene. Both Ca and Ph are highly productive (A.R. Violante, Cutrale, personal communication, https://www.cutrale.com.br/, accessed on 1 March 2023) and may be used either alone or for blending to give a reddish orange colour to usually pale-orange juices such as those from Hamlin and other early and mid-season blond varieties. Further agronomic and nutraceutical studies may help to promote the use of these new mutants potentially beneficial red-fleshed cultivars for the global citrus industry.

## 5. Conclusions

Carotenoid profiles in the fruit pulp of five red-fleshed orange varieties revealed that about 70% were linear carotenoids at all stages of development and maturation. In contrast, the blond varieties accumulated primarily xanthophylls, with *all*-violaxanthin comprising more than 50% of total carotenoids in mature fruit. In this study, the most noteworthy distinction between red-fleshed and blond varieties was the presence of lycopene and the accumulation of β-carotene in red-fleshed ones, which was absent or at traces in blond counterparts. In tropical areas, the incorporation of lycopene-rich red orange juice could be a potential practice to enhance the appeal of orange juices. Among the 5 red-fleshed mutants investigated, both the new Ca and Ph mutants could be used to blend for imparting a reddish-orange colour to typically pale-orange juices, potentially benefiting the global citrus industry. Moreover, the increased accumulation of lycopene in these red-fleshed varieties not only enhances the juice colour but also improves its nutritional value, thanks to lycopene antioxidant properties and increase in β-carotene content.

## Figures and Tables

**Table 1:** Quality parameters from fruits of Valencia (Va), Hamlin (Ha), Pera (Pe), Cara cara (Cc), Mombuca (Mo), Carrancas (Ca), Pinhal (Ph) and Puka (Pk) fruits (*C. sinensis* L. Osbeck) in seasons 1 (2021/2022, up) and 2 (2022/2023, down). The data are means ± SD of at least three independent measurements.

Variety	Total Soluble Solids (°Brix) (TSS)	Titratable Acidity (g/L) (TA)	Maturity Index (MI)	Juice Content Per Orange (JC) (mL)	Fruit Weight (W) (g)	Percentage Juice Content/Fruit Weight (%)	Fruit Diameter (mm)	Fruit Height (mm)	Cortex (mm)	Peel (mm)	Number of Segments	Number of Seed
Valencia	11.7 ± 0.0	0.7 ± 0.3	13.5 ± 0.3	85.0 ± 11.3	219.3 ± 20.2	38.9 ± 4.6	73.7 ± 2.0	76.5 ± 3.1	4.6 ± 0.2	1.7 ± 0.1	10.9 ± 0.3	56.3 ± 7.4
Hamlim	10.8 ± 0.3	0.8 ± 0.0	14.1 ± 0.5	61.3 ± 5.3	168.8 ± 5.1	36.3 ± 3.2	68.9 ± 0.9	69.4 ± 0.6	4.4 ± 0.4	1.6 ± 0.1	10.5 ± 0.2	51.5 ± 6.7
Pera	12.6 ± 0.1	0.7 ± 0.2	12.8 ± 0.3	88.5 ± 1.5	207.7 ± 5.8	42.7 ± 1.8	71.1 ± 0.7	76.1 ± 0.7	5.1 ± 0.1	1.6 ± 0.1	10.6 ± 0.2	61.5 ± 6.4
Cara Cara	9.5 ± 0.0	0.5 ± 0.2	17.9 ± 0.7	38.1 ± 4.5	277.0 ± 26.8	14.4 ± 2.8	86.1 ± 3.8	88.9 ± 1.6	7.5 ± 0.8	2.5 ± 0.4	10.7 ± 0.3	0.3 ± 0.3
Mombuca	8.3 ± 0.0	0.5 ± 0.0	18.0 ± 0.6	92.0 ± 5.3	188.9 ± 8.1	48.6 ± 0.9	71.0 ± 1.2	72.0 ± 1.0	4.6 ± 0.2	1.7 ± 0.1	10.6 ± 0.2	144.3 ± 3.7
Carrancas	7.8 ± 0.0	0.4 ± 0.3	20.1 ± 0.7	55.9 ± 1.6	144.6 ± 7.0	38.7 ± 1.8	64.9 ± 1.3	65.2 ± 1.6	3.2 ± 0.2	1.8 ± 0.1	10.2 ± 0.4	69.0 ± 5.9
Pinhal	8.8 ± 0.3	0.5 ± 0.2	18.9 ± 0.3	54.3 ± 3.1	157.2 ± 2.0	34.5 ± 1.8	65.5 ± 1.0	68.1 ± 1.6	4.9 ± 0.2	2.2 ± 0.2	10.2 ± 0.2	137.5 ± 3.8
Puka	9.6 ± 0.2	0.5 ± 0.1	18.6 ± 0.3	109.1 ± 4.4	209.2 ± 5.4	52.2 ± 2.9	73.3 ± 0.7	72.9 ± 0.9	4.7 ± 0.1	1.6 ± 0.2	10.4 ± 0.3	72.5 ± 9.9
Valencia	11.8 ± 0.3	0.7 ± 0.2	16.1 ± 0.2	92.3 ± 1.9	190.6 ± 4.3	48.4 ± 1.1	70.4 ± 0.8	72.5 ± 0.7	6.1 ± 1.3	1.1 ± 0.1	10.8 ± 0.2	45.0 ± 5.3
Hamlim	9.5 ± 0.1	0.7 ± 0.2	14.5 ± 0.2	80.0 ± 0.6	152.7 ± 2.2	52.4 ± 0.8	66.3 ± 0.7	69.3 ± 1.1	4.4 ± 0.2	2.0 ± 0.0	10.2 ± 0.2	49.3 ± 7.4
Pera	12.6 ± 0.2	0.7 ± 0.3	17.9 ± 0.5	93.8 ± 2.1	181.7 ± 1.2	51.6 ± 0.9	68.2 ± 0.1	73.9 ± 0.7	4.7 ± 0.2	1.6 ± 0.2	9.7 ± 0.2	43.5 ± 6.5
Cara Cara	9.9 ± 0.1	0.5 ± 0.1	18.6 ± 0.4	53.8 ± 3.1	225.7 ± 5.6	27.1 ± 0.7	75.0 ± 0.4	74.9 ± 0.8	6.2 ± 0.3	1.4 ± 0.1	10.4 ± 0.2	7.0 ± 0.4
Mombuca	8.5 ± 0.2	0.5 ± 0.2	17.8 ± 0.4	72.3 ± 1.7	141.2 ± 1.5	51.2 ± 0.8	64.8 ± 0.3	66.8 ± 0.3	5.1 ± 0.1	2.2 ± 0.1	10.4 ± 0.1	147.5 ± 5.5
Carrancas	8.0 ± 0.4	0.4 ± 0.3	20.4 ± 0.8	74.3 ± 6.7	143.9 ± 2.3	51.7 ± 5.2	64.3 ± 0.7	66.0 ± 0.6	4.6 ± 0.2	1.6 ± 0.1	10.4 ± 0.2	89.0 ± 11.7
Pinhal	9.1 ± 0.0	0.5 ± 0.0	19.7 ± 0.1	62.8 ± 2.8	131.0 ± 3.0	47.9 ± 1.3	62.5 ± 0.7	64.5 ± 0.6	4.8 ± 0.2	2.0 ± 0.1	10.4 ± 0.2	116.8 ± 7.8
Puka	9.6 ± 0.3	0.6 ± 0.2	17.1 ± 0.2	66.8 ± 5.6	160.6 ± 6.6	41.9 ± 4.9	74.7 ± 0.6	65.7 ± 0.8	4.4 ± 0.1	1.9 ± 0.1	10.6 ± 0.1	35.5 ± 4.1

## Data Availability

The original contributions presented in the study are included in the article/Supplementary Material, further inquiries can be directed to the corresponding author/s.

## Supplementary Materials

The following are available online at https://www.mdpi.com/article/10.3390/antiox13080994/s1. Table S1. Primer sequences used for the quantitative RT-PCR analyses. Figure S1. Representative climatological condition parameters: (A) temperature, (B) relative humidity and (C) precipitations in different commercial orchards Fazenda Guacho in Sta. Cruz de Rio Pardo (Sao Paulo State, Brazil) during the seasons 2021/22 (season 1) and 2022/23 (season 2). Figure S2. Carotenoid content of phytofluene, lutein and *all*-violaxanthin in the pulp during development and maturation of Valencia (Va), Hamlin (Ha), Pera (Pe), Cara cara (Cc), Mombuca (Mo), Carrancas (Ca), Pinhal (Ph) and Puka (Pk) fruits (*C. sinensis* L. Osbeck) in two different seasons. The samples analysed correspond to the developmental and maturation stages indicated in Figure 2. The physiological fruit stages are indicated: IG (immature-green), MG (mature-green), B (breaker) and FC (full-colour). The data are means ± SD of at least three independent measurements. Statistical analyses were performed using analysis of variance (ANOVA) and an asterisk above the bars indicates significantly different values at *p* ≤ 0.01. Figure S3. Quantitative RT-PCR analysis of the expression of *DXS1*, HDR, HDS and *GGPPS1* genes in the pulp during development and maturation of Valencia (Va), Hamlin (Ha), Pera (Pe), Cara cara (Cc), Mombuca (Mo), Carrancas (Ca), Pinhal (Ph) and Puka (Pk) fruits (*C. sinensis* L. Osbeck) in two different seasons. The samples analysed correspond to the developmental and maturation stages indicated in Figure 2. The physiological fruit stages are indicated: IG (immature-green), MG (mature-green), B bBreaker) and FC (full-colour). Statistical analyses were performed using analysis of variance (ANOVA) and an asterisk above the bars indicates significantly different values at *p* ≤ 0.01. Figure S4. Quantitative RT-PCR analysis of the expression of *ZDS1*, Z*DS2*, *ZDS3*, *ε-LCY2* and *β-LCY1* genes in the pulp during development and maturation of Valencia (Va), Hamlin (Ha), Pera (Pe), Cara cara (Cc), Mombuca (Mo), Carrancas (Ca), Pinhal (Ph) and Puka (Pk) fruits (*C. sinensis* L. Osbeck) in two different seasons. The samples analysed correspond to the developmental and maturation stages indicated in Figure 2. The physiological fruit stages are indicated: IG (immature-green), MG (mature-green), B (breaker) and FC (full-colour). Statistical analyses were performed using analysis of variance (ANOVA) and an asterisk above the bars indicates significantly different values at *p* ≤ 0.01.
