# Supplementary material for: Biochemical Characterization of New Sweet Orange Mutants Rich in Lycopene and β-Carotene Antioxidants
Source: Antioxidants (Basel). 2024 Aug 16;13(8):994. doi: 10.3390/antiox13080994 (PMC11351333; doi:10.3390/antiox13080994)
Supplement: Supplementary file 1 [file antioxidants-13-00994-s001.zip › antioxidants-3103231-supplementary/Supp_Fig.pptx]

## Slide 1
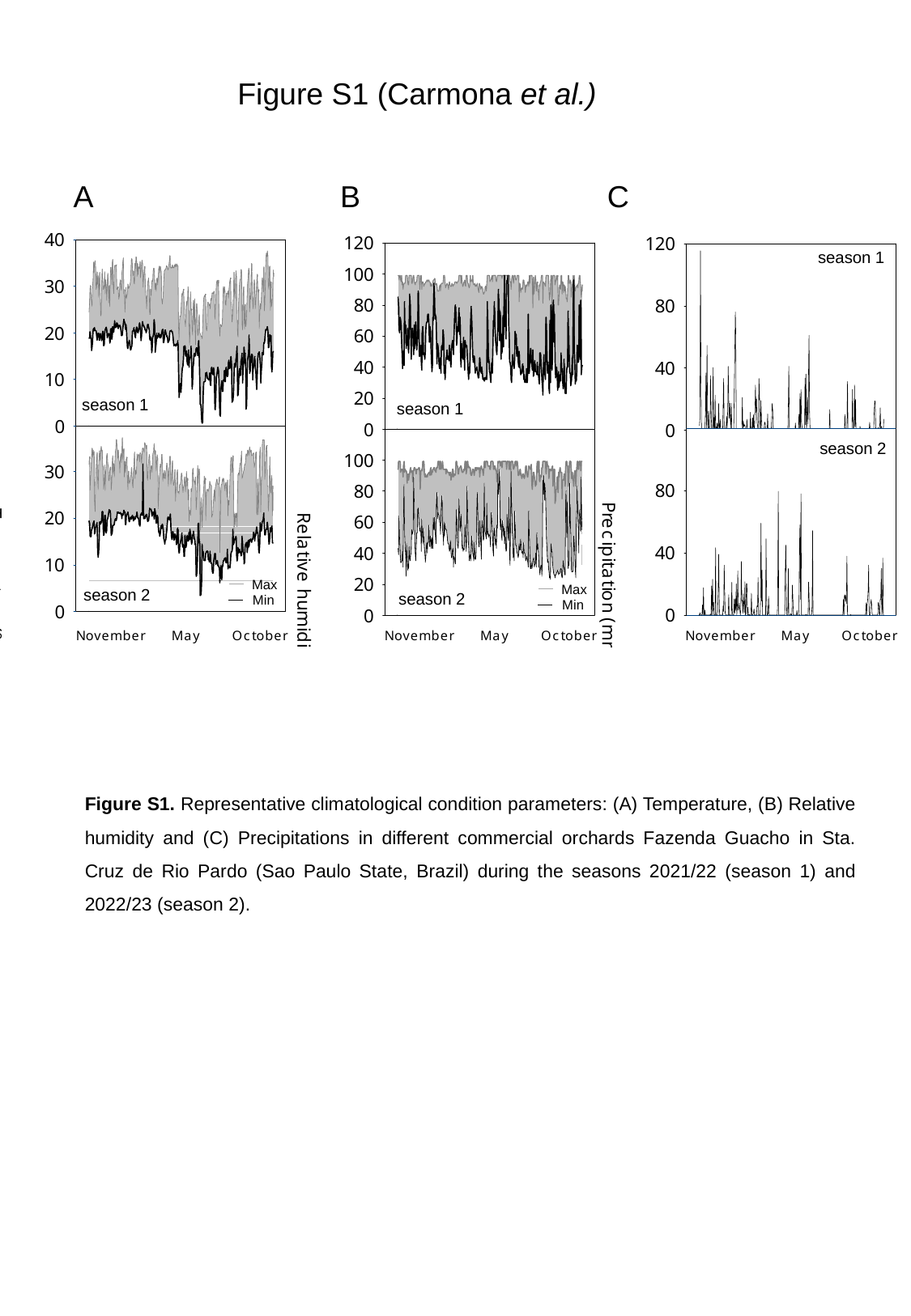

Figure S1 (Carmona et al.)
A
B
C
Max
Min
Max
Min
Figure S1. Representative climatological condition parameters: (A) Temperature, (B) Relative humidity and (C) Precipitations in different commercial orchards Fazenda Guacho in Sta. Cruz de Rio Pardo (Sao Paulo State, Brazil) during the seasons 2021/22 (season 1) and 2022/23 (season 2).

## Slide 2
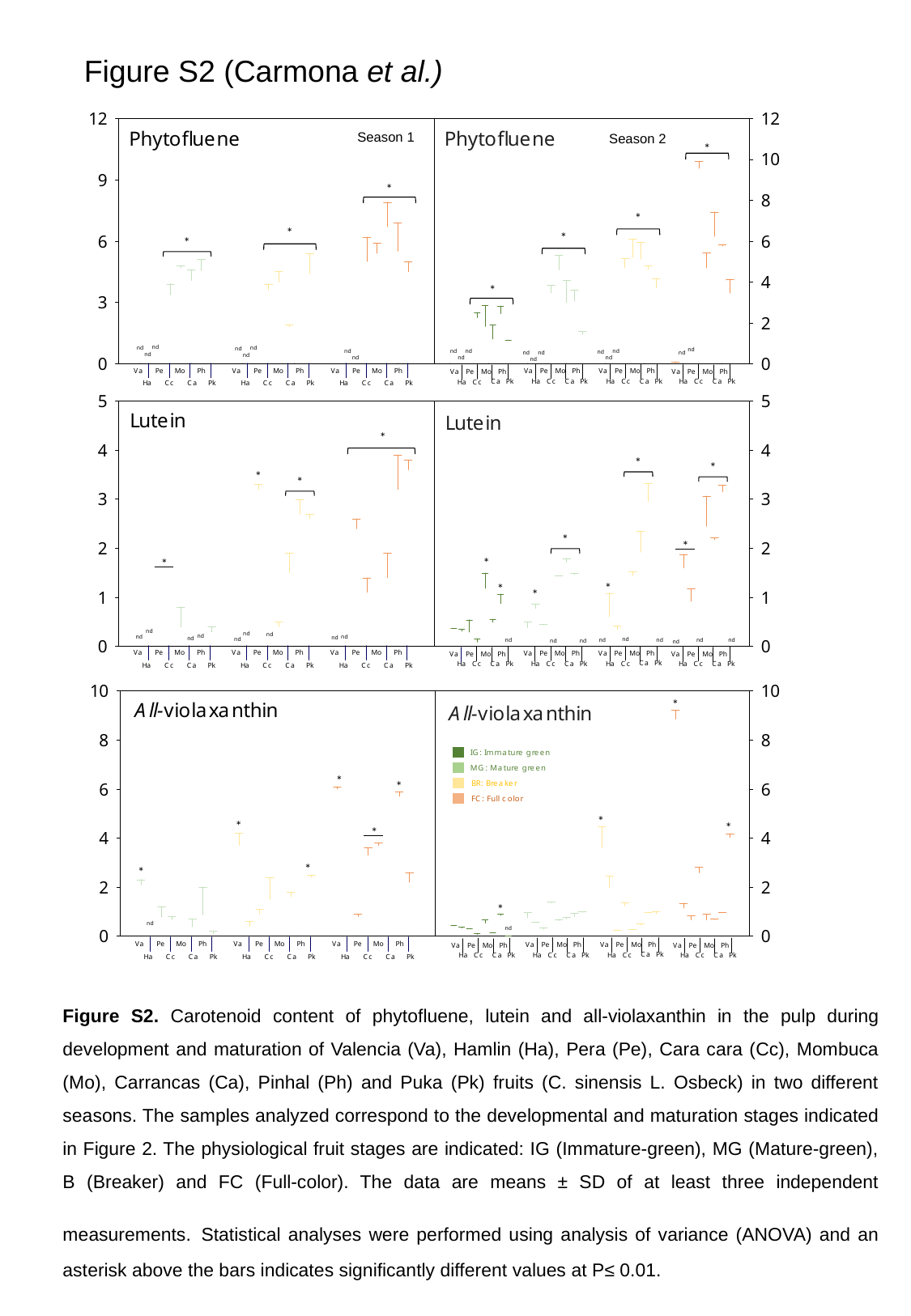

Figure S2 (Carmona et al.)
Season 1
Season 2
*
*
*
*
*
*
*
*
*
*
*
*
*
*
*
*
*
*
*
*
*
*
*
*
*
*
*
*
*
Figure S2. Carotenoid content of phytofluene, lutein and all-violaxanthin in the pulp during development and maturation of Valencia (Va), Hamlin (Ha), Pera (Pe), Cara cara (Cc), Mombuca (Mo), Carrancas (Ca), Pinhal (Ph) and Puka (Pk) fruits (C. sinensis L. Osbeck) in two different seasons. The samples analyzed correspond to the developmental and maturation stages indicated in Figure 2. The physiological fruit stages are indicated: IG (Immature-green), MG (Mature-green), B (Breaker) and FC (Full-color). The data are means ± SD of at least three independent measurements. Statistical analyses were performed using analysis of variance (ANOVA) and an asterisk above the bars indicates significantly different values at P≤ 0.01.

## Slide 3
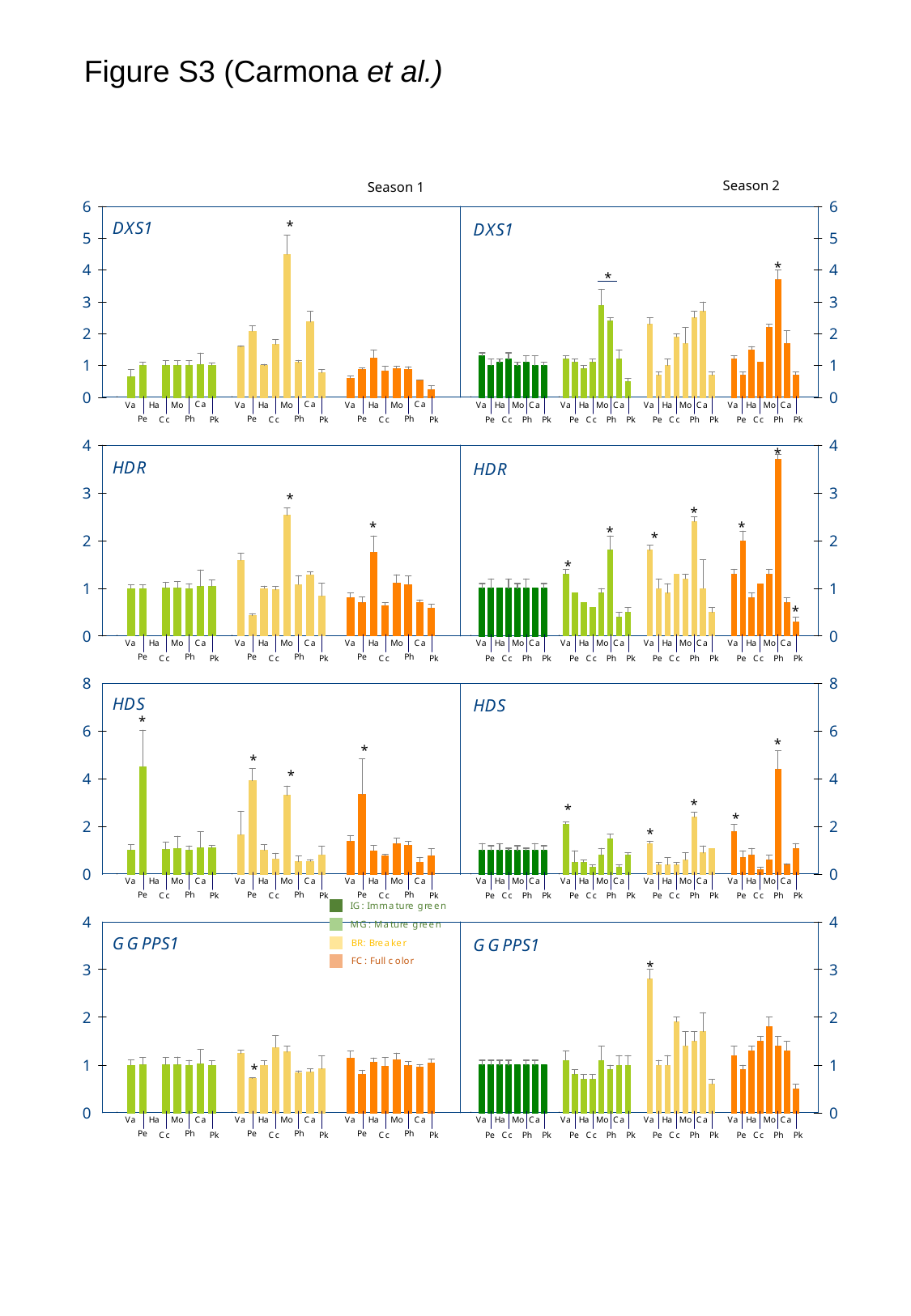

Figure S3 (Carmona et al.)
Season 2
Season 1
*
*
*
*
*
*
*
*
*
*
*
*
*
*
*
*
*
*
*
*
*
*
*

## Slide 4
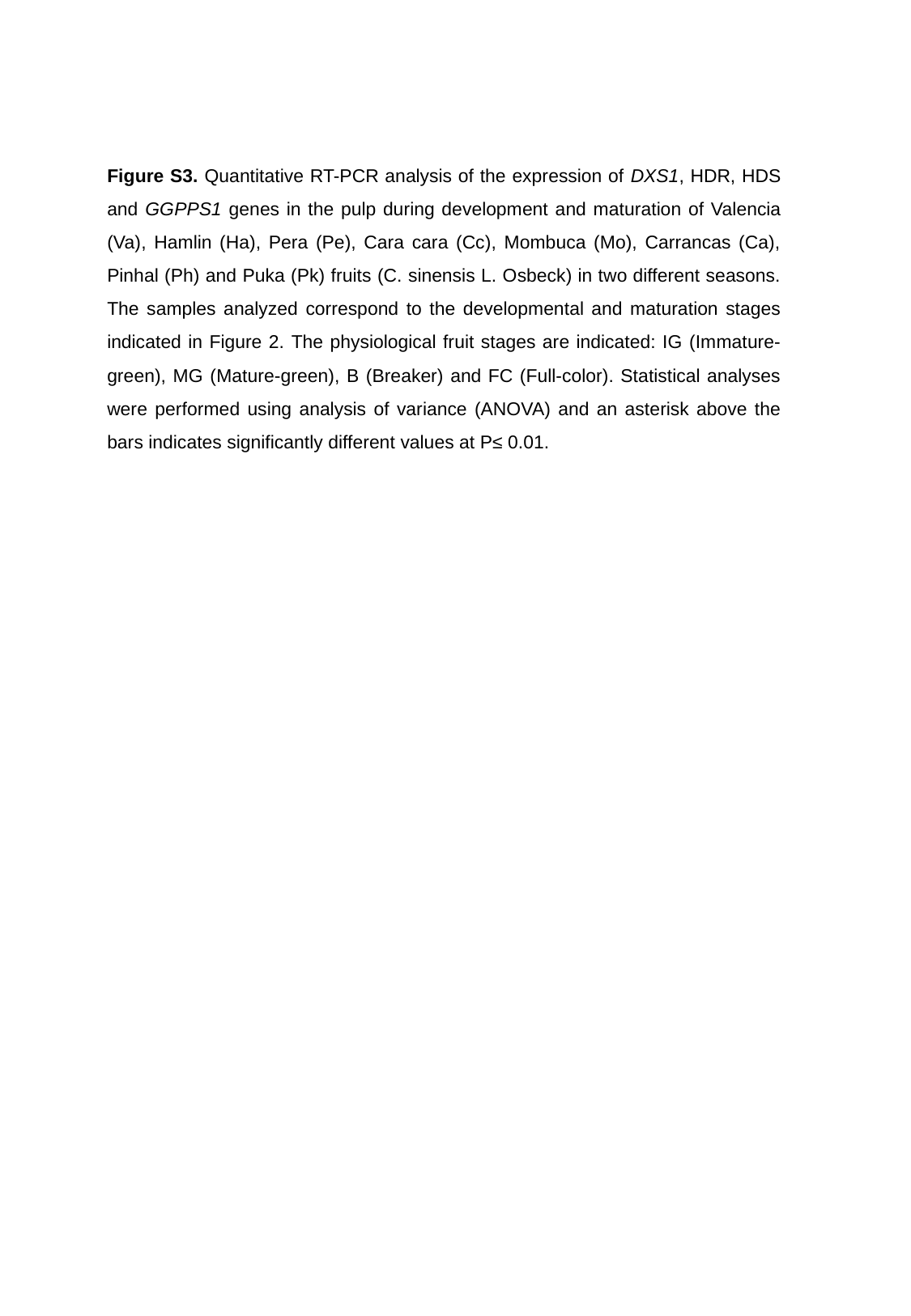

Figure S3. Quantitative RT-PCR analysis of the expression of DXS1, HDR, HDS and GGPPS1 genes in the pulp during development and maturation of Valencia (Va), Hamlin (Ha), Pera (Pe), Cara cara (Cc), Mombuca (Mo), Carrancas (Ca), Pinhal (Ph) and Puka (Pk) fruits (C. sinensis L. Osbeck) in two different seasons. The samples analyzed correspond to the developmental and maturation stages indicated in Figure 2. The physiological fruit stages are indicated: IG (Immature-green), MG (Mature-green), B (Breaker) and FC (Full-color). Statistical analyses were performed using analysis of variance (ANOVA) and an asterisk above the bars indicates significantly different values at P≤ 0.01.

## Slide 5
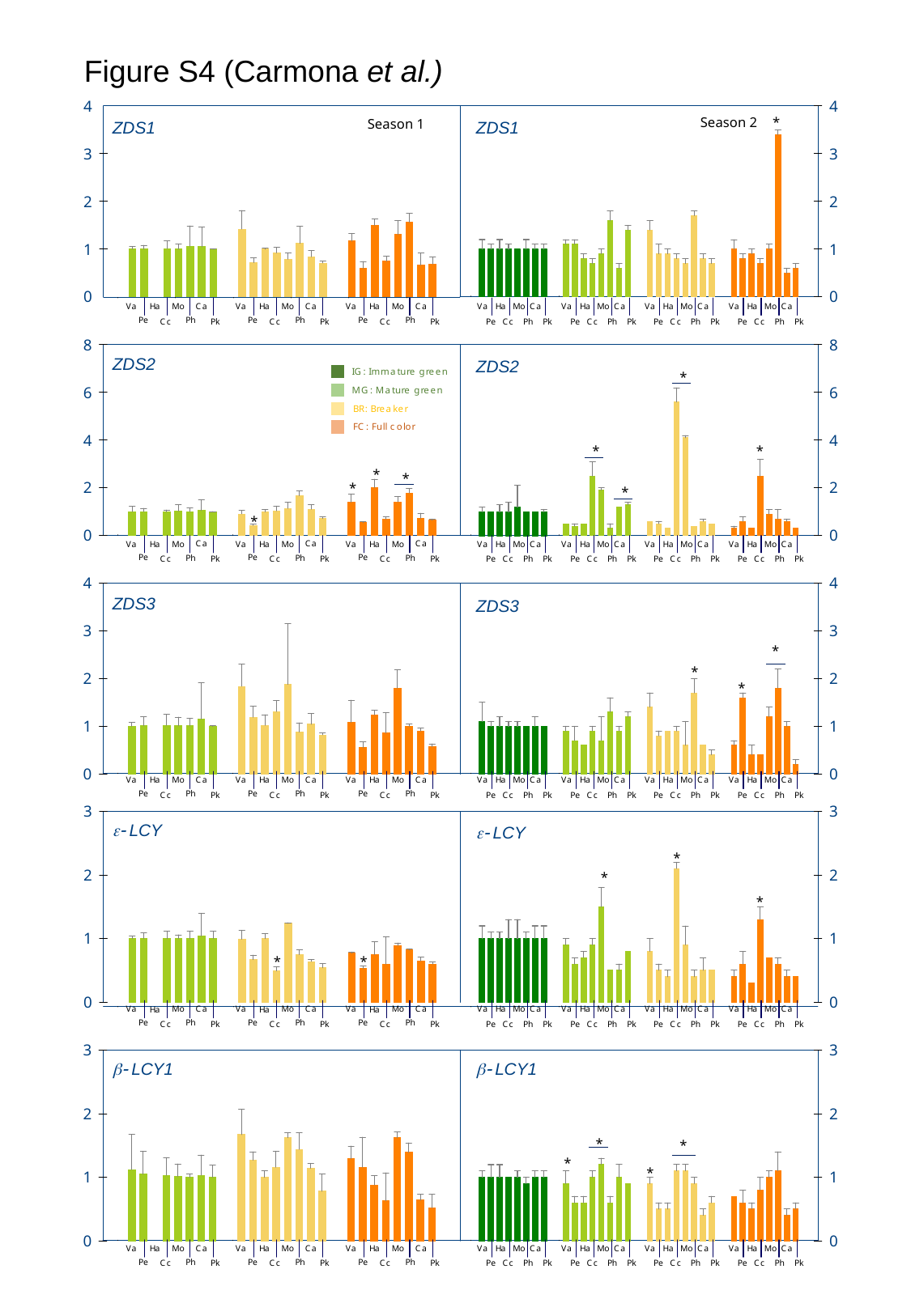

Figure S4 (Carmona et al.)
Season 2
*
Season 1
*
*
*
*
*
*
*
*
*
*
*
*
*
*
*
*
*
*
*
*

## Slide 6
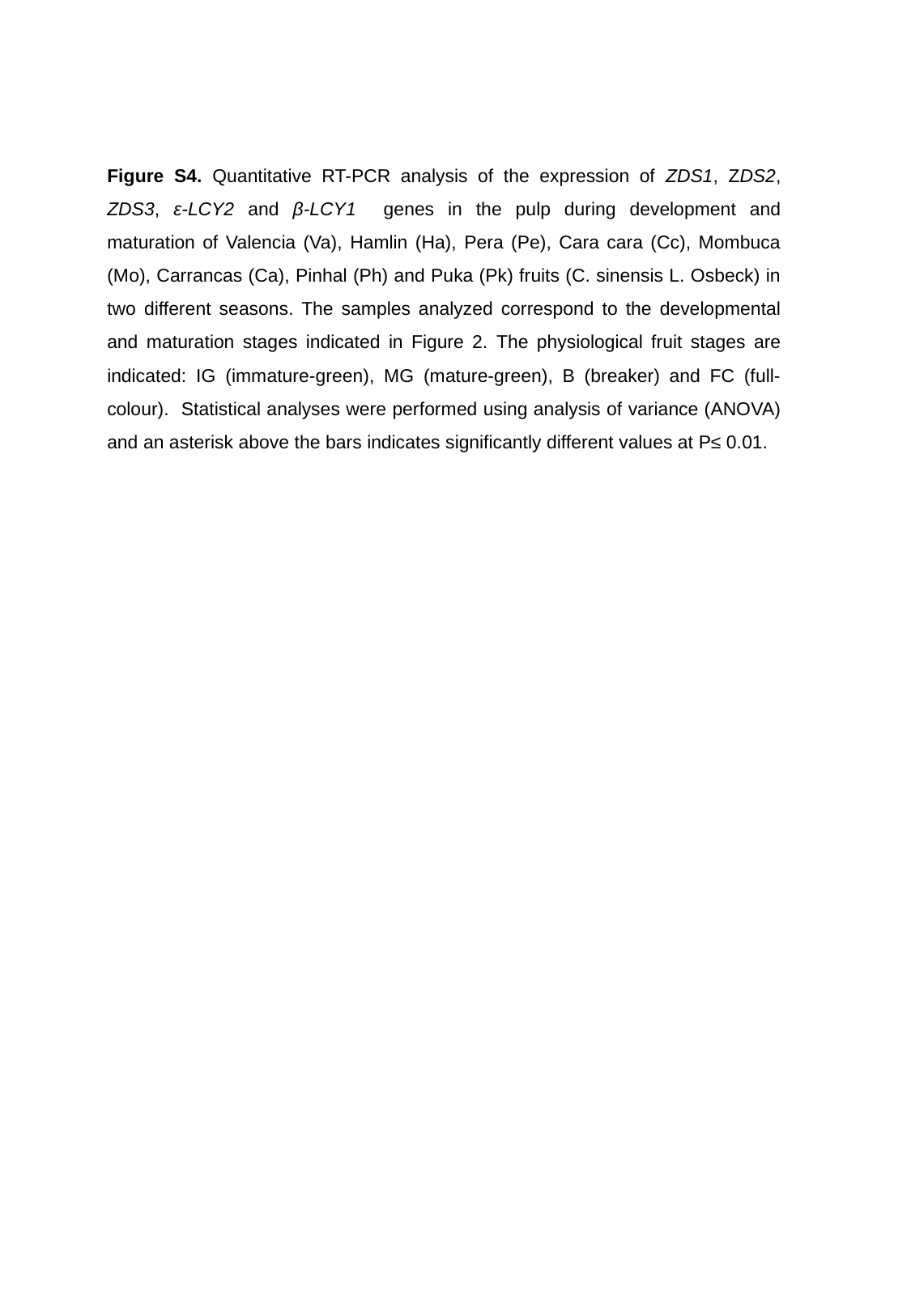

Figure S4. Quantitative RT-PCR analysis of the expression of ZDS1, ZDS2, ZDS3, ε-LCY2 and β-LCY1 genes in the pulp during development and maturation of Valencia (Va), Hamlin (Ha), Pera (Pe), Cara cara (Cc), Mombuca (Mo), Carrancas (Ca), Pinhal (Ph) and Puka (Pk) fruits (C. sinensis L. Osbeck) in two different seasons. The samples analyzed correspond to the developmental and maturation stages indicated in Figure 2. The physiological fruit stages are indicated: IG (immature-green), MG (mature-green), B (breaker) and FC (full-colour). Statistical analyses were performed using analysis of variance (ANOVA) and an asterisk above the bars indicates significantly different values at P≤ 0.01.

## Slide 7
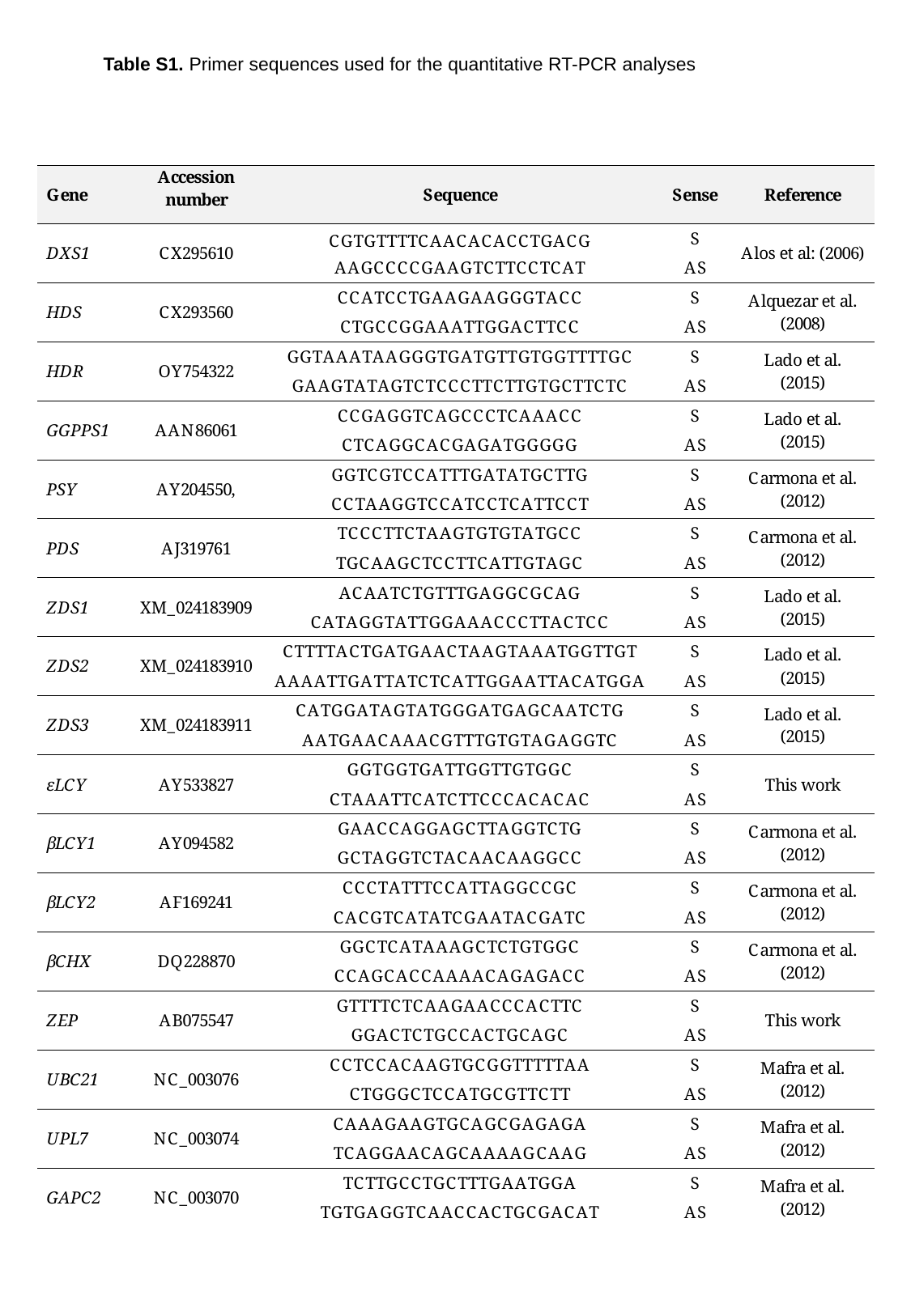

Table S1. Primer sequences used for the quantitative RT-PCR analyses
